# Is There a Link between the Molecular Basis of Juvenile Idiopathic Arthritis and Autoimmune Diseases? Systematic Review

**DOI:** 10.3390/ijms25052803

**Published:** 2024-02-28

**Authors:** Ignacio Ventura, Gemma Clara Meira-Blanco, María Ester Legidos-García, Marcelino Pérez-Bermejo, María Teresa Murillo-Llorente

**Affiliations:** 1 Molecular and Mitochondrial Medicine Research Group, School of Medicine and Health Sciences, Catholic University of Valencia San Vicente Mártir, C/Quevedo no. 2, 46001 Valencia, Spain; ignacio.ventura@ucv.es; 2 Translational Research Center San Alberto Magno CITSAM, Catholic University of Valencia San Vicente Mártir, C/Quevedo no. 2, 46001 Valencia, Spain; 3 School of Medicine and Health Sciences, Catholic University of Valencia San Vicente Mártir, C/Quevedo no. 2, 46001 Valencia, Spain; gemmaclara.meira@mail.ucv.es; 4 SONEV Research Group, Faculty of Medicine and Health Sciences, Catholic University of Valencia San Vicente Mártir, C/Quevedo no. 2, 46001 Valencia, Spain; ester.legidos@ucv.es (M.E.L.-G.); mt.murillo@ucv.es (M.T.M.-L.)

**Keywords:** anti-nuclear antibodies, antibiotics, non-bacterial osteomyelitis, cyclooxygenase, diabetes mellitus, haploinsufficiency, glycated haemoglobin, interferon

## Abstract

Juvenile Idiopathic Arthritis (JIA) is currently the most common chronic rheumatic disease in children. It is known to have no single identity, but a variety of diagnoses. Under-diagnosis is a barrier to early treatment and reduced complications of the disease. Other immune-mediated diseases may coexist in the same patient, making research in this area relevant. The main objective was to analyse whether links could be established between the molecular basis of JIA and other immune-mediated diseases. Early diagnosis may benefit patients with JIA, which in most cases goes undetected, leading to under-diagnosis, which can have a negative impact on children affected by the disease as they grow up. Methods: We performed a PRISMA systematic review focusing on immune molecules present in different autoimmune diseases. Results: A total of 13 papers from different countries dealing with the molecular basis of JIA and other immune diseases were evaluated and reviewed. Conclusions: Most of the autoimmune diseases analysed responded to the same group of drugs. Unfortunately, the reason for the under-diagnosis of these diseases remains unknown, as no evidence has been found to correlate the immunomolecular basis with the under-diagnosis of these immune-mediated diseases. The lack of information in this area means that further research is needed in order to provide a sound basis for preventing the development of immune-mediated diseases, especially in children, and to improve their quality of life through early diagnosis and treatment.

## 1. Introduction

Juvenile Idiopathic Arthritis (JIA) is an immune-mediated disease affecting children under sixteen for at least six weeks. It manifests with joint inflammation, stiffness, and restricted movement. Unlike adult rheumatoid arthritis, JIA is often outgrown, but it may impact bone development in those still growing [1,2].

The ILAR (International League of Associations for Rheumatology) classifies JIA into seven categories that often manifest with distinctive symptoms and help guide treatment. These are systemic juvenile arthritis, oligoarticular JIA, polyarticular JIA, psoriatic arthritis, enthesitis-related arthritis (ERA), and undifferentiated arthritis, the diagnosis of which requires the individual to have no condition or to meet criteria for more than one category [3,4,5,6].

JIA is currently the most common chronic rheumatological disorder in childhood. Females tend to be more affected, although no gender differences are found in systemic JIA and males are more affected in JIA enthesitis. Incidence and prevalence can vary, possibly attributed to under-diagnosis. The estimated overall incidence ranges from 1.6 to 23 per 100,000 children under sixteen years annually. The prevalence is approximately 3.8 to 400 per 100,000 [7]. Oligoarticular JIA is the most prevalent, followed by the polyarticular form, with the psoriatic type being the least common diagnosis. Efforts have been made to link environmental factors, including vaccination, breastfeeding, or trauma, to various JIA categories. However, limited correlation has been established between the environment and JIA, with genetic predisposition being a more significant factor [7]. Studies indicate a higher susceptibility for boys compared to girls, and the peak age for JIA development is reported to be 2–3 years [8]. In addition, other underlying risk factors for many diseases, such as prenatal maternal smoking, hospitalisation during the first year of life or day care during the first six years of life, have not been found to be associated with the development of JIA [9].

The treatment objective is not curative but aims to support children with JIA in sustaining a dignified, socially active, and physically engaged quality of life. This involves employing a combination of strategies to alleviate inflammation and pain while preserving joint movement and strength. Early initiation of treatment is essential to optimize individualized responses based on factors such as age, JIA type, onset, and severity, striving for the most effective outcomes [10].

As a first step, non-steroidal anti-inflammatory drugs are prescribed as a symptomatic treatment, inhibiting cyclooxygenase (COX) and interfering with prostaglandin synthesis [11]. The second step will be to add corticosteroids, which are anti-inflammatory drugs with immunosuppressive capacities, which may be the therapy of choice in severe cases with systemic effects [11,12]. The prescription of disease-modifying antirheumatic drugs is the third step in the treatment of JIA. The drug of choice is methotrexate, acting as a remission inducer and establishing the primary therapeutic axis in JIA patients. The fourth and final step on the therapeutic ladder involves biologic agents, which have specific actions against cells of the inflammatory response and are intended for hospital use only [13].

Currently, under-diagnosis of JIA is a barrier to treatment and research. The fact that it is a childhood disease is relevant when thinking about treatment, due to its characteristics to be considered [7]. The prognosis is not as optimistic as initially perceived. In numerous cases, patients do not achieve remission, and the disease persists for years, significantly impacting the quality of life of affected children. Effective treatment necessitates multidisciplinary collaboration to stabilise and enhance clinical manifestations [14]. Children are rarely affected by immune disorders, but when they are, early treatment can improve quality of life and prognosis and prevent irreversible damage in adulthood. Early diagnosis can therefore benefit patients with JIA, which in most cases goes undetected, leading to under-diagnosis, which can have a negative impact on children affected by the disease as they grow up. The main aim of this work was to analyse the possible relationship between the molecular basis of JIA and other immune-mediated diseases, and to explore the molecular basis that may be involved in JIA. Understanding the immunopathogenesis of Juvenile Idiopathic Arthritis (JIA) necessitates a comprehensive examination of key immunological parameters. Antinuclear Antibodies (ANA) have been identified as potential markers, signifying their association with autoimmune responses in JIA. Concurrently, investigations into the Major Histocompatibility Complex (MHC) reveal its role in influencing the genetic predisposition to JIA. Tumour Necrosis Factor Alpha (TNF-α), an inflammatory mediator, is implicated in the pathogenesis of JIA, elucidating its involvement in the inflammatory cascade. Elevated levels of S100 proteins, correlated with inflammation, further emphasize the intricate immune response in JIA. Additionally, the exploration of various interleukins, including IL-1 and IL-6, underscores their potential significance in the disease process. In summary, the multifaceted nature of JIA is unravelled by scrutinizing these immunological parameters, offering valuable insights into the disease’s aetiology and paving the way for targeted therapeutic interventions.

### 1.1. Immune Bases

#### 1.1.1. ANA

ANAs are autologous immunoglobulins that target cells and cytoplasmic components linked to rheumatic diseases. Despite their heightened sensitivity, ANAs lack specificity for any particular rheumatic disease and are detected in roughly 100% of rheumatic patients and 3–15% of the healthy population. Due to this lack of specificity, ANAs are not a valuable diagnostic parameter but serve a limited role in confirming the presence of an autoimmune disease [15].

#### 1.1.2. Major Histocompatibility Complex (MHC)

The human leukocyte antigen (HLA) system plays a crucial role in the immune system. Governed by genes on chromosome six, this system encodes surface molecules responsible for presenting antigenic peptides to T-cell receptors. It consists of two main classes: class I and class II. In MHC I, transmembrane glycoprotein molecules are located on the surface of nucleated cells. They comprise an alpha heavy chain linked to a beta2 microglobulin through two binding domains, with the heavy chain encoded by the HLA-A, HLA-B, and HLA-C genes. TCD8+ cells, often possessing a cytotoxic function, interact with this type of major histocompatibility complex and are capable of recognizing infected cells [15]. MHC II molecules are located on antigen-presenting cells, activated T cells, or cells induced by interferon gamma. These molecules consist of two polypeptide chains, alpha and beta, each with two domains: one peptide-binding and one Ig-like. These chains are encoded by genes such as HLA-DP, -DQ, or -DR. T cells responding to MHC II molecules typically express CD4 and function as helper cells. The HLA-B27 allele, found in MHC type I, is linked to numerous autoimmune diseases. In JIA, it may be present in patients with enthesitis-type JIA and is strongly associated with ankylosing spondylitis. Therefore, it is plausible that patients with this JIA subtype may develop spondylitis over time. However, HLA-B27 is also present in 5–15% of the general population, limiting its specificity as a diagnostic test [16].

#### 1.1.3. Tumour Necrosis Factor (TNF-α)

Tumour necrosis factor (TNF) is an inflammatory cytokine produced by macrophages and monocytes during acute inflammation. It plays a crucial role in signalling events that lead to necrosis or apoptosis, making it significant in infection and cancer resistance. TNF-α primarily exerts its effects by binding to cell membrane receptors with a molecular weight of 55 kDa or 75 kDa. A distinctive feature of TNF is its extracellular domain, comprising 2–6 cysteine-rich repeats. Additionally, structurally related “decoy receptors” complementarily bind to TNF molecules, rescuing cells from apoptosis [17]. TNF-α is implicated in both the psoriatic and enthesitic forms of JIA, where an abnormal synthesis of inflammatory cytokines occurs in the synovial membrane. It plays a crucial role in initiating, maintaining, and destroying synovial tissue by expressing pro-inflammatory genes like IL-6, IL-1, and IL-18. These cytokines activate fibroblasts, leading to the synthesis of matrix metalloproteases that degrade cartilage, and osteoclasts, causing complete destruction of joint architecture. In systemic lupus erythematosus (SLE), TNF-α’s role remains controversial, yet it is associated with higher levels of autoantibodies and kidney damage. In type 1 diabetes mellitus (DM1), TNF-α acts cytotoxically in pancreatic islets, inducing β-cell apoptosis and inhibiting insulin secretion. Additionally, the presence of dendritic cells and macrophages in the early stages of diabetes has been linked to β-cell inflammation [17].

#### 1.1.4. S100 Proteins

The S100 proteins, a family of twenty-four cytosolic calcium-binding proteins, are distributed across intracellular, extracellular, and regulatory domains. Apart from their roles in adaptive immunity, tissue development, and repair, S100 proteins play a crucial part in regulating proliferation, differentiation, Ca^2+^ homeostasis, and inflammation [18]. Specifically implicated in inflammation-mediated responses, these proteins are released into an acellular compartment during cellular stress or inflammation. They then bind to surface receptors, activating intracellular signalling pathways associated with cell migration, proliferation, apoptosis, or inflammation [19]. Activation of S100 proteins is observed in systemic JIA and LSE, making their determination valuable in differentiating other febrile and autoinflammatory syndromes.

#### 1.1.5. Interleukins (IL)

Interleukins (ILs) are cytokines expressed not only by leukocytes, as initially believed, but also by various other cell types. Their significance lies in the differentiation and activation of immune cells, encompassing both pro-inflammatory and anti-inflammatory properties. Interleukins play pivotal roles in the activation of inflammatory or immune processes, exhibiting both paracrine and autocrine functions. Additionally, they frequently impact the synthesis and actions of other interleukins [20,21].

**Figure 1:**
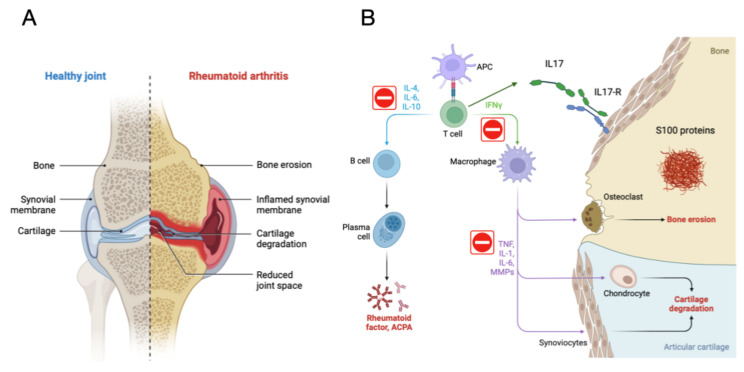
Aetiopathogenesis and Molecules Involved in JIA. Juvenile Idiopathic Arthritis (JIA) stands out as the most prevalent chronic arthritis in children, characterised by joint inflammation and pain. Key molecules in the pathogenesis of JIA are identified: Tumour Necrosis Factor-alpha (TNFα), Interleukin 17 (IL-17), S100 Proteins in serum. Medications for the treatment of JIA and other autoimmune diseases are described, highlighting TNF-α Inhibitors. In the figure, potentially interesting pathways to block are indicated with a red circle. In the specific context of Juvenile Idiopathic Arthritis (JIA), the receptor for IL-17 is found in various immune system cells, including T cells, macrophages, neutrophils, and fibroblasts. Activation of this receptor by IL-17 promotes inflammation and the production of inflammatory cells, contributing to the development and progression of JIA. In the inflamed joints of children with JIA, S100 proteins are deposited, secreted by inflammatory cells such as macrophages and neutrophils. These deposits occur in the articular cartilage, synovial membrane, and synovial fluid, contributing to cartilage loss and joint pain. Elevated levels of S100 proteins in blood and joints are used for the diagnosis and monitoring of JIA and other inflammatory diseases. (**A**) show the joint of a healthy individual compared to a person with JIA, and (**B**) show the cellular mechanism involved in JIA. Created by authors.

Figure 1 discusses the aetiopathogenesis and key molecules involved in JIA. It also indicates potentially interesting pathways to block, highlighted with a red circle. In the specific context of JIA, it is highlighted that the receptor for IL-17 is found on various cells of the immune system and that its activation by IL-17 promotes inflammation and the production of inflammatory cells, thus contributing to the development and progression of JIA. In the inflamed joints of children with JIA, S100 proteins secreted by inflammatory cells such as macrophages and neutrophils are deposited. These deposits contribute to cartilage loss and joint pain and are found in the articular cartilage, synovial membrane, and synovial fluid.

## 2. Materials and Methods

Preferred Reporting Items for Systematic Reviews and Meta-Analysis (PRISMA) criteria were used for this systematic review [22] which was registered in the records of the Open Science Framework (https://doi.org/10.17605/OSF.IO/J264G; accessed on 24 January 2024). A literature search of PubMed and Web of Science databases was performed between December 2022 and April 2023.

### 2.1. Search Strategy

Search strategies were crafted by combining free text in the abstract and title fields with controlled vocabulary from the medical thesaurus (MESH). Terms used include: “JIA”, “juvenile arthritis”, “idiopathic juvenile arthritis”, “children”, “juvenile chronic arthritis”, “juvenile rheumatoid arthritis”, “silent disease”, “underdiagnosis”, “leukocyte antigens”, “histocompatibility”, “HLA antigens”, “human leukocyte antigens”, “cell growth factor”, “IL-6 receptor”, “IL-6”, “IL-18”, “IL-18 receptor”, “TCGF receptor”, “helper cells”, “helper inducer T-lymphocytes”, “helper T-cells”, “helper inducer T cells”, “calcium-binding protein”, “vitamin D-dependent calcium-binding protein”, “lymphotoxin”, “lymphotoxin alpha”, “tumour necrosis factor beta”, “soluble lymphotoxin alpha”, “ANA”, “antinuclear antibody “anti-DNA antibodies”, “antinuclear antibodies”, “Libman’s disease”, “uveitis”, “diabetes”, “ankylosing spondylarthritis”, “chronic lymphocytic thyroiditis”, “Hashimoto’s thyroiditis”, “Hashimoto’s syndrome”. Terms were amalgamated using Boolean operators (AND) and (OR). The choice of these keywords was based on the need to address specific aspects related to JIA and its interaction with other diseases, thus facilitating a more comprehensive exploration of the relevant literature in the proposed research area.

In addition, to include papers that may have been overlooked and to obtain a larger number of articles on the topic, a manual search of the bibliographic references of the included studies was carried out.

### 2.2. Data Extraction

An initial screening of titles and abstracts was followed by a full-text review of eligible studies. Articles on JIA or immunological diseases of interest, published in English or Spanish, were eligible for inclusion. Non-original reports, reviews, case reports, conference abstracts, editorials, commentaries, studies including patients older than 16 years, or articles not contributing to or complementing the study objectives were excluded.

### 2.3. Quality Assessment

To assess study design and quality, the Joanna Briggs Institute (JBI) checklists [23] appropriate for each type of study were used. These checklists have 4 response options for all items: Yes, No, Unclear, or Not applicable. Evidence was scored using the number of Yes responses out of the total possible responses, expressed as a percentage, and scoring below 50% was considered low quality. If a result was not applicable in any of the items, the total score would have been reduced by as many points as there were items that were not applicable in the study being assessed. This was not the case in any of the reviewed studies, where no items were scored as not applicable. On the basis of their quality, no eligible studies were excluded from the review. For each study, the quality assessment was reviewed independently by two authors: I.V. and M.P.-B. Interrater reliability was high, and any disagreements were discussed between G.M.-B., M.E.L.-G, and M.T.M.-L until agreement was achieved.

## 3. Results

**Figure 2:**
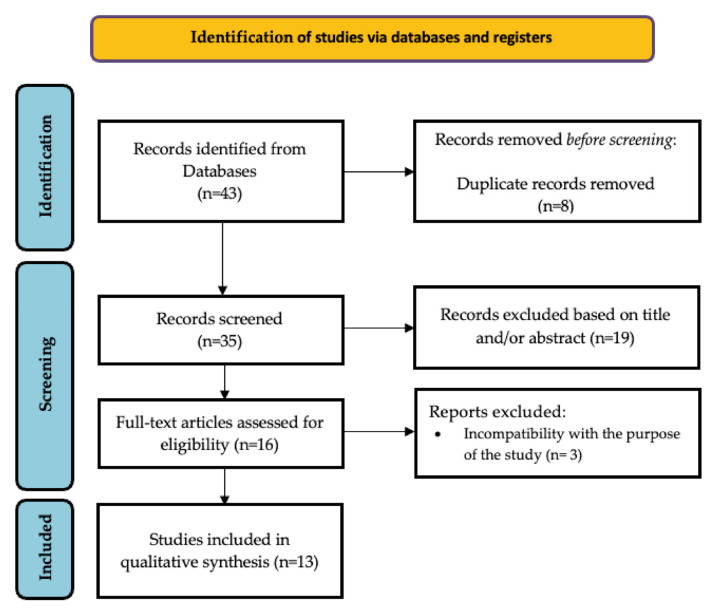
PRISMA flowchart of study selection process.

As shown in the search diagram (Figure 2), 43 articles were retrieved. After excluding duplicates, 35 articles remained. 19 articles were excluded after title reading, and 16 articles were retained for full text review. Any that did not meet the inclusion criteria were excluded. Finally, 13 articles were included in this systematic review. Table 1 shows the main characteristics of each study and Supplementary Tables S1–S3 show the quality assessment of the studies.

All included papers discuss JIA alongside other immunological diseases, including Hashimoto’s, SLE, spondylitis, uveitis, and diabetes. References to immune molecules and/or markers or allusions to treatments are also included. Of the thirteen articles included, four are cohort study, three cross-sectional study and six case and control study. The main countries were the USA and Germany with two studies each. China, Russia, Thailand, South Korea, France, Finland, and India contributed one study each and there were two multi-country studies.

## 4. Discussion

Upon initiating this systematic review, our inquiry delved into the existence of underlying molecular bases in JIA that could potentially link with other immune diseases, potentially rendering children more susceptible to developing additional conditions. Our aim was also to investigate whether early diagnosis and intervention could mitigate the onset of other immune disorders and their associated outcomes. However, the data gleaned from the reviewed articles did not yield conclusive findings, as disparities in results among different papers surfaced. Despite this, certain similarities emerged, aligning with our initial hypothesis.

While not explicitly delving into the molecular basis, Räisänen et al. [32] shed light on the increased likelihood of developing immune diseases in cases of preterm birth and neonatal antibiotic use. These factors may impede the establishment of a robust autoimmune system in children, potentially triggering aberrations such as self-aggregating cells or immune cell dysregulation. This, in turn, could lead to prolonged inflammation and autoimmune intolerance, eventually culminating in the manifestation of immune diseases. Additionally, their work highlighted the role of genetic mutations and dysregulation in inducing immune dysfunction, resulting in TNF-α mutations. Such mutations could disrupt the inhibition of cytokine production, paving the way for an overwhelming inflammatory response, persistent inflammation, recruitment of inflammatory cells, and the initiation of autoimmune diseases.

It is crucial to emphasize the absence of specific biomarkers for these diseases. As observed in the majority of the reviewed articles, biomarkers like S100 proteins or the cross-activation of cytokines (IL-17, IL-18, IL-6) are evident across various autoimmune diseases. These shared biomarkers may suggest a potential connection to an underlying pathogenesis.

HLA-B27+ and ANA+ patients generally exhibited a higher susceptibility to immune diseases compared to non-HLA-B27+ individuals [25,29,31,33]. However, it is essential to recognize that these markers are not specific predictors for the onset of autoimmune disease, given their presence in a high percentage of the general population. Consequently, while they may exhibit some association with autoimmune disease, further genetic research is imperative for identifying concealed mutations that could lead to the onset of autoimmune conditions. Notably, ANA+ JIA patients have displayed an augmented response to LPS stimulation [31], suggesting an elevated autoimmune response and an increased propensity for uveitis, indicating an underlying crosstalk or chemokinetic activation.

Moreover, despite HLA-B27 positivity being prevalent in various autoimmune diseases, it has been demonstrated to decrease the risk of uveitis post JIA diagnosis [24]. However, the significance of this finding remains uncertain, as only this study (with limitations in recruitment and sample size) reported a reduced risk associated with HLA-B27.

The findings regarding S100 proteins in the studies exhibit variations, rendering them inconclusive and even contradictory. Kostik et al. [27] report elevated levels of S100A8/A9 proteins in JIA patients, while Angeles-Han et al. [28] demonstrate no difference between a control group and patients with disease, raising concerns about the reliability of the results. Conversely, another study highlights consistently elevated levels of S100A12 proteins, present in several autoimmune diseases, in patients with active uveitis underlying JIA. These elevated levels may prove useful in categorizing uveitis activity in affected children [24]. Notably, S100A12 acts as a neutrophil regulator, and its impairment may perpetuate cytokine activation. However, this study found no difference in serum S100A12 levels between patients without uveitis and those with active uveitis at follow-up visits, contrary to another paper [34] indicating the presence of both S100A8/A9 and S100A12 in patients with systemic JIA but not uveitis. Additionally, S100A8/A9+ patients exhibited a greater need for biologic therapy, conflicting with other studies that showed a better response to MTX. The conflicting results across different papers underscore the unreliability of S100 proteins in elucidating common hidden pathways in immune diseases.

Regarding interleukins, Harms et al. [26] observed elevated IL-18 levels in a patient with various autoimmune diseases, along with elevated IL-17A and IL-7, potentially contributing to inflammation and disease progression. A lack of a specific chemokine response was also noted. Conversely, another study [27] reported lower cytokine levels in patients with DM1 compared to those with CNO, although still higher than in control patients. IL levels were considered to play a crucial underlying role in both diseases. Additional studies [28,31] demonstrated increased levels of IL-8 and IL-6, peaking at 6 h after LPS stimulation. The involvement of IL-8 in neutrophil-induced inflammation may aid in differentiating active immune-mediated uveitis from uveitis of other aetiologies.

Similarly, Parida et al. [33] noted elevated levels of IL-6 in patients with RS, alongside elevated IL-17, and although distinct, they observed comparable cytokine profiles in peripheral spondyloarthritis. Additionally, IL-18 levels were found to be higher in systemic JIA than in other subtypes. Interestingly, patients not undergoing drug treatment displayed heightened levels of IL-6 and IL-17. IL-6 plays a pivotal role in promoting Th17 and inhibiting regulatory T cells, potentially leading to TNF-α expression and the promotion of inflammation, although it has not been conclusively deemed deterministic.

While these observations may appear coincidental, they could signify intrinsic immune pathways that, when activated, render immunocompromised patients more susceptible to various diseases. Although these studies reveal increased levels of the same cytokines in different immune disorders, further research is imperative to unravel the intricate relationships between them. Nevertheless, it seems plausible that an underlying link exists. A common thread in these articles is the recognition that ILs play a known role in activating the inflammatory cascade. When dysregulated, they have the potential to perpetuate inflammation and contribute to disease development, establishing them as crucial players in these disorders and valuable biomarkers.

TNF-α emerged as a recurring theme in the reviewed articles. While Harms et al. [26] did not show elevated levels, they could not detect it in controls or DM1. Conversely, Wang et al. [31] demonstrated a peak after LPS stimulation in JIA-related uveitis and an association with HA20. This association may lead to negative feedback inhibition of NF-kB, triggering B-cell and interleukin proliferation, inducing inflammation, and potentially leading to various autoimmune diseases [24]. Higher TNF-α levels were found in acute RS and UPSA compared to controls. However, chronic patients exhibited lower levels of TNF-α, likely attributed to the chronic nature of inflammatory activation. Drug-naive JIA patients [35] also displayed higher TNF-α levels (which potentiate TH17 activity and inflammation) than treated patients. These elevations in untreated patients seem to be linked to the effects of JIA drugs on inflammation.

Lerkvaleekul et al. [36] analysed differences in lymphocyte subpopulations in SLE and their long-term outcomes, discovering that elevated CD4+ levels increased the likelihood of developing arthritis. An imbalance of lymphocyte subtypes altered the immune response in SLE patients, triggering an inflammatory response. Wang et al. [31] also highlighted the pivotal role of CD4+ helper cells in Th17 activation and the inflammatory cascade in JIA patients. Another study [26] identified CD4+ cells as a strong risk factor for the development of DM1, suggesting a potential connection among these three diseases.

Finally, treatment efficacy is critical to the quality of life of patients with immune disorders. Etanercept, an anti-TNF-alpha drug, was effective not only in JIA but also in DM1 [25,29]. Also, anti-TNF therapy was more common in patients with immune-mediated uveitis and JIA and was useful in reducing the incidence of uveitis and preventing visual impairment as well as in treating JIA [25,29]. In addition, MTX has been shown to reduce the risk of autoimmune uveitis and other complications in previously treated patients with JIA [24]. Monoclonal antibodies such as infliximab have shown efficacy in the treatment of JIA, spondyloarthritis, and sacroiliitis [35,36]. This high rate of positive results in several immune-mediated diseases makes it important to explore this area in terms of therapeutic targets.

Related work is limited and has important limitations, which further complicates the study of these relationships. The consideration of demographic factors is crucial to contextualize and better understand immunological variables in the study of Juvenile Idiopathic Arthritis (JIA). Various demographic factors, such as age, gender, and socioeconomic environment, may influence the manifestation and progression of JIA. It is crucial to explore how these demographic variables can impact the statistical significance of the investigated immunological parameters. For instance, age at diagnosis and disease duration could play a key role in immune response and treatment efficacy. Additionally, ethnic and geographic diversity should be considered, as there might be variations in the prevalence and expression of immunological biomarkers in different populations. Integrating these demographic considerations into the analysis will strengthen the external validity of the results and provide a more comprehensive understanding of the relationship between immunological factors and demographic characteristics in JIA.

Regarding the strengths of the study, the comprehensiveness in reviewing the scientific literature on the relationship between immunological factors and JIA is noteworthy. The inclusion of diverse studies and exploration of multiple biomarkers provides a comprehensive view of the complexity of the disease. However, it is essential to acknowledge certain limitations. Differences in outcomes among the reviewed studies may stem from variations in research methods, studied populations, and other potential biases. Moreover, heterogeneity in therapeutic approaches and a lack of standardization in measuring some biomarkers may affect result comparability.

Regarding generalisability, the diversity of studied populations and the inclusion of multiple immunological diseases strengthen the applicability of findings to a broader range of patients. Nonetheless, caution should be exercised when extrapolating results to specific clinical contexts. In summary, addressing these strengths and limitations will contribute to a more complete and balanced interpretation of results and ensure the appropriate applicability of conclusions to clinical practice.

## 5. Conclusions

In conclusion, this systematic review, which sought to determine whether there is a link between the molecular basis of juvenile idiopathic arthritis (JIA) and other immune diseases, did not definitively establish a direct link. While the possibility of a relationship seems plausible, the current evidence suggests that other immune diseases may more likely manifest in the presence of JIA rather than sharing a common molecular basis. The majority of autoimmune diseases discussed in the literature exhibited favourable responses to Methotrexate (MTX) or other Disease-Modifying Antirheumatic Drugs (DMARDs), hinting at potential interconnections within the immune system. However, the under-diagnosis of these diseases remains unexplained, lacking evidence correlating immunomolecular bases with the under-diagnosis of immune-mediated conditions. The gaps in knowledge underscore the need for further research to establish a robust foundation for preventing the development of immune-mediated diseases, particularly in children. Early diagnosis and treatment stand as crucial components for improving the quality of life in affected individuals.

## Figures and Tables

**Table 1:** Main characteristics of each study analysed.

Study	Year	Sample Size	Country	Study Type	Main Findings
[24]	2018	*n* = 954	Germany	Descriptive multicentric	This study outlined that female sex, early onset of JIA, and being oligoarthritic and ANA-positive favoured the development of a uveitis flare. Treatment with methotrexate (TMTX) was found to reduce the risk of uveitis flares. Mean S100A12 levels remained similar in patients with or without uveitis. Uveitis-related complications did not show significant differences based on sex, ANA positivity, JIA category, or age at onset. Patients treated with disease-modifying antirheumatic drugs (DMARDs) such as methotrexate or biologic DMARDs prior to uveitis had slightly fewer uveitis-related complications in the initial uveitis registry, including a lower risk of uveitis onset. A lower risk of uveitis in JIA patients was observed in HLA-B27+ children, despite the presence of HLA-B27 also being associated with uveitis.
[25]	2019	*n* = 147	France	Descriptive retrospective bicentric	In the current study, the predominant diagnosis was JIA associated with uveitis, often presenting as bilateral and anterior cases. When linked with JIA, they predominantly exhibited ANA positivity. The study indicated that a substantial number of patients achieved uveitis inactivity following treatment with MTX or biologic therapy. Complications were more prevalent in ANA-positive patients, underscoring the challenges of early diagnosis and emphasizing the necessity for further research in this area. Disease-modifying antirheumatic drugs (DMARDs), particularly anti-TNF-alpha antibodies, demonstrated favourable outcomes in the majority of cases and exhibited potential in preventing visual impairment. Early intervention in immune-mediated diseases correlated with fewer visual complications and an improved visual prognosis.
[26]	2020	*n* = 63	USA	Case-control	The study assessed the cytokines IL-17A, IL-7, and IL-18, with the latter associated with hyperglycaemia and being one of the most abundant interleukins. Elevated levels of these cytokines were observed in patients with DM1, while TNF-alpha was undetected in both groups. However, there were no increased levels of IL-18 when comparing controls and DM1 patients, suggesting a lack of evidence for a specific immune response. The results indicate a general absence of a distinctive cytokine response specific to DM1 patients.
[27]	2021	*n* = 117	Russia	Case-control	In this study, cytokine levels of S100A8/A9 protein, IL-4, IL-17, IL-18, IL-1, and TNF-alpha were assessed in patients with NBOC, JIA, and DM1, as well as in healthy controls. Patients with NBOC exhibited elevated levels of interleukins compared to controls and DM1 patients. Conversely, TNF-alpha and S100A8/A9 protein levels were higher in patients with JIA.
[28]	2021	*n* = 40	USA	Case-control	In this study, measurements were taken for S100A8/A9, IL-2, IL-18, IL-8, and TNF-gamma. No statistically significant differences in S100A8/A9 levels were observed between controls and patients with JIA-related uveitis. However, among children with JIA-related uveitis, S100A12 was found to be increased in active compared to inactive uveitis. Furthermore, when comparing uveitis by activity index, patients with active uveitis exhibited higher levels of S100A12 protein and IL-8.
[29]	2021	*n* = 5529	Multiplecountries	Cohort study	This study revealed a lower utilization of anti-IL treatment in patients who developed uveitis, while the use of anti-TNF therapy was higher in those with uveitis compared to those without. Despite HLA-B27 and ANA-positive patients being more prone to uveitis development, their lack of specificity rendered them ineffective in determining uveitis risk. Although HLA-B27 is frequently linked to anterior uveitis, its presence in the general population limits its utility in identifying JIA patients at risk of uveitis. Interestingly, undifferentiated arthritis did not associate with a reduced likelihood of uveitis, potentially due to the cohort’s ANA and HLA-B27 positive status.
[30]	2021	*n* = 155	South Korea	Descriptive	This study concluded that the autoimmune diseases most strongly associated with uveitis are JIA, accounting for 45.5% of all systemic JIA cases (14.8% of all systemic cases). Other diseases include Behçet’s disease (6.5%), Kawasaki’s disease (1.9%), Vogt-Koyanagi-Harada syndrome (1.9%), and tubulo-intestinal nephritis (1.3%). Unilateral and anterior uveitis emerged as the most common anatomical types. Uveitis cases linked with inflammatory diseases exhibited a significantly higher rate of systemic treatment. HLA-B27 and ANA levels were higher in patients with JIA compared to those with idiopathic uveitis. In Korea, children with uveitis have a 33% chance of having an underlying immune disease, with JIA being the most prevalent.
[31]	2021	*n* = 30	China	Descriptive	In this study, peaks in monocyte, TNF-alpha, and IL-6 were measured six hours after LPS stimulation. ANA titres exhibited a decrease following the control of ocular inflammation. TNF-alpha and IL-6 demonstrated rapid increases across all groups, followed by a decline after the six-hour peak. The ANA-positive group displayed a heightened cytokine response to LPS, indicating a robust reaction, while the ANA-negative group exhibited a slower response. The ANA-positive group had a delayed response, whereas the control group demonstrated a steady response. Additionally, the study highlighted the significance of TLR4 signalling in macrophages for acute uveitis development. An association between MHC class II and JIA was identified. Synovial fluid from JIA patients with inflamed joints exhibited elevated levels of Th17, correlating with arthritis severity. This investigation underscored that younger age and ANA-positive status are risk factors for uveitis development. However, the study emphasized that ANA is not considered a specific marker for uveitis in JIA patients.
[32]	2021	*n* = 11,407	Finland	Cohort study	In this study, 2.1% of the recruited adolescents received diagnoses of JIA, DM1, thyroiditis, and inflammatory bowel disease. The development of immune disorders was most significantly associated with preterm birth, which can hinder the transfer of intrauterine antibodies from the female parent, and postnatal antibiotic use. Caesarean sections were not found to be linked to an increased risk of autoimmune diseases. However, among preterm children born by caesarean section, there was a higher incidence of postnatal antibiotic use compared to those born via vaginal delivery. Nevertheless, this antibiotic use appears to be more closely related to the onset of autoimmune diseases rather than the caesarean section itself.
[33]	2021	*n* = 100 (Cohort I) *n* = 38 (Cohort II)	India	Cohort study	This study identified two distinct groups: In cohort I, HLA-B27 was present in over 80% of patients with RS and UPSA, with subtype B*2705 being the most prevalent. In cohort II, cytokine levels were measured, revealing no significant differences between the two groups. When comparing chronic and acute RS, lower levels of TNF-alpha and higher levels of IL-6 and IL-17 were observed in chronic patients, as well as in children with UPSA or RS, compared to healthy children. No differences in HLA-B27 were found in either group.
[34]	2021	*n* = 266	Germany	Descriptive multicentric	In this study, biomarkers such as IL-18, S100A8/A9, and S100A12 proteins exhibited elevated levels in systemic JIA compared to other categories. Interestingly, baseline cytokine levels were higher in patients with oligoarthritis, despite the expectation that the extended type would display greater inflammatory activity. Notably, higher levels of S100A8/A9 proteins were observed in those treated with disease-modifying drugs, establishing them as biomarkers for children requiring biologic therapy. This contrasts with other studies showing that elevated S100A8/A9 protein levels are linked to a better response to MTX. Patients not receiving drug treatment displayed higher levels of IL-6, IL-17, and TNF-α, suggesting increased Th17 activity. Those with active JIA showed a stronger association with heightened inflammation levels in the early stages of the disease, indicating a connection with the severe and persistent form of the condition.
[35]	2021	*n* = 902	Multiplecountries	Descriptive	In this study, children diagnosed with enthesitis-related arthritis (ERA) tended to be older at the initial diagnosis and were more likely to test positive for HLA-B27. In both conditions, the majority of children experienced polyarticular involvement at some stage. Those with sacroiliitis were more commonly treated with biologics, as DMARDs typically prove ineffective in patients with axial disease. Interestingly, contrary to findings in other studies, HLA-B27 was present in a lower percentage of patients in this study.
[36]	2022	*n* = 102	Thailand	Cohort study	The high γ, δ T cell group and the elevated NK cell group were significantly correlated with a higher frequency of mucosal ulcers, while the high CD4+ T cell group showed a significant association with a higher frequency of arthritis. The evidence suggests that an imbalance in lymphocyte subtypes plays a role in the immune response of SLE patients, contributing to the inflammatory response through interferon I, II, and NK cells. These cells produce cytokines responsible for regulating T, B, and NK cells. It’s noteworthy that not all LSE patients exhibit the expected response to B-cell targeted therapy. γ δ T cells and NK cells have been linked to oral ulcers, CD8+ cells to vasculitis, and CD4+ cells to arthritis.

ANA: Antinuclear antibody; CD4: Linfocite, cluster of quadruple differentiation; DM1: Diabetes Mellitus 1; DMARDs: Disease-modifying antirheumatic drugs; ERA: Juvenile psoriasic arthritis; HLA-B27: human leukocyte antigen B*27; IL: Interleukins; JIA: Juvenile idiopathic arthritis; MTX: methotrexate; NBOC: non-bacterial osteomyelitis; NK: Natural killer cells; RS: Reiter’s syndrome; TLR4: Protein receiver type Toll 4; Th17: Type of white globule; TNF-Alpha: Tumour necrosis factor alpha; TNF-Gamma: Interferon gamma expression; UPSA: Undifferentiated peripheral spondylarthritis; B*2705: Specific variant of the B27 allele (one of the variants of HLA-B27); CD8+: Cytotoxic T cells.

## Data Availability

Not applicable.

## Supplementary Materials

The following supporting information can be downloaded at: https://www.mdpi.com/article/10.3390/ijms25052803/s1.
