# Supplementary material for: Is There a Link between the Molecular Basis of Juvenile Idiopathic Arthritis and Autoimmune Diseases? Systematic Review
Source: Int J Mol Sci. 2024 Feb 28;25(5):2803. doi: 10.3390/ijms25052803 (PMC10931547; doi:10.3390/ijms25052803)
Supplement: Supplementary file 1 [file ijms-25-02803-s001.zip › ijms-2895781-supplementary.pdf]

Is there a link between the molecular basis of juvenile idiopathic arthritis and autoimmune diseases? Supplementary material

Ignacio Ventura, Gemma Clara Meira-Blanco, María Ester Legidos-García, Marcelino Pérez-Bermejo and María Teresa Murillo-Llorente

Table S1. Studies appraised using the Joanna Briggs Institute critical appraisal checklist for case control studies

	Were the criteria for inclusion in the sample clearly defined?	Were the study subjects and the setting described in detail?	Was the exposure measured in a valid and reliable way?	Were objective, standard criteria used for measurement of the condition?	Were confounding factors identified?	Were strategies to deal with confounding factors stated?	Were the outcomes measured in a valid and reliable way?	Was appropriate statistical analysis used?	Score out of 8 (100%)
Shin et al., 2021 [28]	Y	Y	Y	Y	U	Y	Y	Y	7 (87.5%)
Morelle et al., 2021 [29]	Y	Y	Y	Y	N	U	Y	Y	6 (75%)
Wang et al., 2021 [30]	Y	Y	Y	Y	Y	Y	Y	Y	8 (100%)
Tappeiner et al., 2018 [31]	Y	Y	Y	Y	Y	Y	Y	Y	8 (100%)
Ganeya et al., 2020 [35]	Y	N	Y	Y	Y	Y	Y	Y	7 (87.5%)
Rumsey et al., 2021 [36]	Y	U	Y	Y	U	Y	N	Y	5 (62.5%)

Y: Yes; N: No; U: Unclear; NA: Not applicable

Table S2. Studies appraised using the Joanna Briggs Institute critical appraisal checklist for analytical cross-sectional studies

	Were the groups comparable other than the presence of disease in cases or the absence of disease in controls?	Were cases and controls matched appropriately?	Were the same criteria used for identification of cases and controls?	Was exposure measured in a standard, valid and reliable way?	Was exposure measured in the same way for cases and controls?	Were confounding factors identified?	Were strategies to deal with confounding factors stated?	Were outcomes assessed in a standard, valid and reliable way for cases and controls?	Was the exposure period of interest long enough to be meaningful?	Was appropriate statistical analysis used?	Score out of 10 (100%)
Harms et al., 2020 [24]	Y	Y	Y	Y	Y	Y	Y	Y	U	Y	9 (90%)
Kostik et al., 2021 [25]	Y	Y	U	Y	U	Y	Y	Y	Y	Y	8 (80%)
Angeles et al., 2021 [26]	Y	Y	Y	U	U	Y	N	Y	Y	Y	7 (70%)

Y: Yes; N: No; U: Unclear; NA: Not applicable

Table S3. Studies appraised using the Joanna Briggs Institute critical appraisal checklist for cohort studies

	Were the two groups similar and recruited from the same population?	Were the exposures measured similarly to assign people to both exposed and unexposed groups?	Was the exposure measured in a valid and reliable way?	Were confounding factors identified?	Were strategies to deal with confounding factors stated?	Were the groups/participants free of the outcome at the start of the study (or at the moment of exposure)?	Were the outcomes measured in a valid and reliable way?	Was the follow up time reported and sufficient to be long enough for outcomes to occur?	Was follow up complete, and if not, were the reasons to loss to follow up described and explored?	Were strategies to address incomplete follow up utilized?	Was appropriate statistical analysis used?	Score out of 11 (100%)
Van Straalen et al., 2021 [27]	Y	Y	Y	U	Y	Y	Y	Y	Y	U	N	8 (72.7%)
Lerkvaleekul et al., 2022 [32]	Y	Y	Y	Y	U	Y	Y	Y	N	U	Y	8 (72.7%)
Räisänen et al., 2021 [33]	Y	Y	Y	Y	Y	Y	Y	Y	Y	Y	Y	11 (100%)
Parida et al., 2021 [34]	Y	Y	Y	Y	U	Y	Y	Y	U	U	Y	8 (72.7%)

Y: Yes; N: No; U: Unclear; NA: Not applicable
